# Physical Activity, Body Composition, and Cardiometabolic Health during Pregnancy: A Compositional Data Approach

**DOI:** 10.1249/MSS.0000000000002996

**Published:** 2022-09-03

**Authors:** JOHANNA SANDBORG, JAIRO H. MIGUELES, EMMIE SÖDERSTRÖM, MARIE BLOMBERG, PONTUS HENRIKSSON, MARIE LÖF

**Affiliations:** 1 Department of Biosciences and Nutrition, Karolinska Institutet, Huddinge, Stockholm, SWEDEN; 2 Department of Health, Medicine and Caring Sciences, Linköping University, Linköping, SWEDEN; 3 PROFITH (PROmoting FITness and Health through physical activity) Research Group, Department of Physical Education and Sports, Faculty of Sport Sciences, Research Institute of Sport and Health, University of Granada, Granada, SPAIN; 4 Department of Obstetrics and Gynecology and Department of Biomedical and Clinical Sciences, Linköping University, Linköping, SWEDEN

**Keywords:** COMPOSITIONAL DATA ANALYSIS, ACCELEROMETRY, CARDIOVASCULAR HEALTH, BODY FATNESS, GESTATIONAL WEIGHT GAIN, GESTATION

## Abstract

**Purpose:**

The aim of this study was to examine the cross-sectional and longitudinal associations of 24-h movement behaviors (sleep, sedentary behavior (SB), light physical activity (LPA), and moderate-to-vigorous physical activity (MVPA)) with body composition and cardiometabolic health in i) early and ii) late pregnancy (gestational weeks 14 and 37).

**Methods:**

This observational study utilized cross-sectional (*n* = 273) and longitudinal data (*n* = 242) from the HealthyMoms trial. Time spent in movement behaviors over seven consecutive 24-h periods (ActiGraph wGT3x-BT accelerometer), body composition (Bod Pod), and cardiometabolic health indicators (glucose levels, homeostatic model for insulin resistance (HOMA-IR), systolic and diastolic blood pressure, metabolic syndrome (MetS) score) were measured in early and late pregnancy.

**Results:**

In early pregnancy, reallocating time to MVPA from LPA, SB, and sleep was associated with lower MetS score (adjusted *γ* = −0.343, *P* = 0.002). Correspondingly, reallocating time to LPA from SB and sleep in early pregnancy was associated with lower body weight (adjusted *γ* = −5.959, *P* = 0.047) and HOMA-IR (adjusted *γ* = −0.557, *P* = 0.031) at the same time point. Furthermore, reallocating time to LPA from SB and sleep in early pregnancy was associated with lower fat mass index (adjusted *γ* = −0.668, *P* = 0.028), glucose levels (adjusted *γ* = −0.315, *P* = 0.006), HOMA-IR (adjusted *γ* = −0.779, *P* = 0.004), and MetS score (adjusted *γ* = −0.470, *P* = 0.027) in late pregnancy. The changes in behaviors throughout pregnancy were not associated with body weight, body composition, and MetS score in late pregnancy.

**Conclusions:**

Our results demonstrated that increasing LPA or MVPA while reducing SB and sleep was associated with lower weight and more favorable cardiometabolic health in early pregnancy. In contrast, LPA in early pregnancy seems to be a stimulus of enough intensity to improve body composition and cardiometabolic health indicators in late pregnancy.

The prevalence of excessive gestational weight gain and the rates of associated pregnancy complications (e.g., gestational diabetes, preeclampsia, gestational hypertension, and newborn macrosomia) have risen over the last decades (1–3). Alarmingly, around half of pregnant women in high-income countries exceed the recommendations for an optimal gestational weight gain (4,5). Lifestyle factors such as unhealthy diet and low levels of physical activity during pregnancy contribute to the current negative trend. At the same time, these factors offer opportunities to intervene. To illustrate, women reporting higher levels of physical activity (e.g., reaching the recommended level of 150 min of moderate intensity physical activity per week) have been found to have a lower risk of excessive gestational weight gain and less pregnancy complications, and thus more optimal maternal and fetal health (6,7).

The World Health Organization published new global guidelines for physical activity and sedentary behavior (SB) in November 2020, which, for the first time, included recommendations for pregnant women (8). Although it is well established that physical activity in pregnancy is beneficial (e.g., decreased risk of preeclampsia, gestational hypertension, gestational diabetes, excessive gestational weight gain, and delivery complications), little is known on the associations of dosage and levels of physical activity and pregnancy outcomes, and more research in this area has been requested (8). To illustrate, although the World Health Organization recommends pregnant women to accumulate 150 min of moderate physical activity per week and to limit the time spent being sedentary, the certainty of the evidence for these recommendations is classified as moderate and low, respectively (8). In that context, it is also relevant to highlight that physical activity tends to decrease during pregnancy, and most women do not reach the aforementioned recommendations as pregnancy advances. In fact, in a recent Swedish study (*n* = 2203), only 27% of the women reported reaching the recommendations in the third trimester (6). Similarly, studies in pregnant women using objective measures (i.e., accelerometry) have shown low adherence to the recommendations (9–11). Moreover, it is unclear how activity levels (i.e., SB, light physical activity (LPA), and moderate-to-vigorous physical activity (MVPA)) change in pregnancy and how much of a decline in physical activity can still provide health benefits.

In recent years, the 24-h continuum, which considers movement behaviors (i.e., sleep, SB, LPA, and MVPA) as dependent of each other, has become more recognized (12). In comparison to isotemporal substitution models, which have traditionally been used to investigate time reallocation across behaviors (13) and linear regression models, compositional data analysis is not affected by multicollinearity. Previous research has shown beneficial effects of physical activity of higher intensity levels (e.g., MVPA) on insulin resistance, diastolic blood pressure, HDL, and the risk of excessive gestational weight gain (14–16). However, these studies have not accounted for the co-dependency of movement behaviors, which may produce spurious findings as it is impossible to increase the daily time in one behavior while maintaining the rest constant. Pregnancy is often characterized by physiological changes and potential pregnancy complications (e.g., pelvic girdle pain), which alter most women’s ability to maintain the same level of, for example, MVPA throughout pregnancy, and thus, this might be especially important in pregnancy.

The aim of this study was to examine associations of movement behaviors (i.e., sleep, SB, LPA, and MVPA) in early pregnancy (gestational week 14) with body composition and cardiometabolic health in i) early pregnancy and ii) late pregnancy (gestational week 37) using compositional data analysis. Furthermore, we investigated whether changes in the behaviors from early to late pregnancy (gestational weeks 14–37) were associated with body composition and cardiometabolic health in gestational week 37.

## METHODS

### Study design and participants

This study utilizes cross-sectional and longitudinal data from the HealthyMoms randomized controlled trial (ClinicalTrials.gov; NCT03298555), which has been described in more detail previously (17,18). In short, the trial (conducted between October 2017 and November 2020) investigated the effectiveness of a 6-month smartphone intervention (the HealthyMoms app) on gestational weight gain (primary outcome), diet, and physical activity (secondary outcomes; *n* = 305). Women 18 yr or older, pregnant with a singleton fetus, literate in Swedish, and with no previous eating disorder or medical conditions attending maternity clinics in Östergötland were eligible for participation. Participants were recruited in the first trimester, and outcome measures were assessed in early (gestational week 14) and late pregnancy (gestational week 37). All measurements have been described in detail previously (17–19). In brief, these included measurements of, for example, anthropometrics (i.e., weight, height, body composition), cardiometabolic health indicators (i.e., blood pressure, blood lipids, glucose, and insulin levels), objective measures of movement behaviors (wGT3x-BT accelerometer; ActiGraph, Pensacola, FL), and demographics (e.g., age, parity, educational level, birth country, prepregnancy body mass index (BMI)). As described previously (18), a small number of women (early pregnancy, *n* = 23; late pregnancy, *n* = 18) were not able to wear the accelerometer on the wrist because of health restrictions at their workplace, and these women were excluded from the analyses. Participants with valid accelerometer data in early pregnancy (*n* = 273) were included in the cross-sectional analyses, and participants with valid data at both time points (gestational weeks 14 and 37) were included in the longitudinal analyses (*n* = 242). The HealthyMoms trial was approved by the Regional Ethical Review Board in Linköping, Sweden (reference numbers 2017/112-31 and 2018/262-32), and all participants provided written informed consent before entering the trial. The study is reported according to the Strengthening The Reporting of OBservational Studies in Epidemiology checklist (20).

### Movement behaviors

Physical activity, SB, and sleep were measured using the wrist-worn ActiGraph wGT3x-BT accelerometer (ActiGraph) for seven consecutive 24-h periods (at all times with the exception of water activities). To assist the sleep-detection algorithm, women were instructed to report their everyday in bed and out of bedtime. The accelerometer was programmed to collect raw acceleration at 100 Hz. Raw accelerations were then aggregated as the Euclidean Norm of the raw accelerations with negative values rounded to zero in 5-s epochs. Appropriate thresholds were used to identify SB and physical activity intensities (i.e., 35 mg for SB, 35–100 mg for LPA; 100 mg for MVPA) (21,22). Data analysis and inclusion followed the same criteria as in our previous publication reporting the main outcomes from the effectiveness trial (18), with at least one valid day of data and a valid day defined as at least two-thirds of the 24-h period being wear time and at least two-thirds of the wake time being wear time. Daily average sleep, SB, LPA, and MVPA were calculated as the mean of weekdays and weekend days, and data were processed in the software program R and the package GGIR (version 2.1-3) (23) (the configuration file with the GGIR processing specifications can be found in Supplemental Digital Content 1, http://links.lww.com/MSS/C704).

### Body composition

A wall-stadiometer (Tillquist, Spånga, Sweden) was used to measure body height. Body weight and body composition were measured using Bod Pod (COSMED) as described previously (19,24). In short, this method enables calculation of body density by accurately measuring body volume and body weight (measured when only wearing underwear) and using the two-component model (i.e., dividing the body into fat mass and fat-free mass) (25,26). Furthermore, body composition was derived by using gestational week appropriate densities for fat mass and fat-free mass (26). BMI was calculated as weight (in kilograms) divided by height squared (meter squared). Similarly, fat mass index (FMI) and fat-free mass index was calculated as fat mass (in kilograms) or fat-free mass (in kilograms) divided by height squared (in meter squared), respectively.

### Cardiometabolic health

As described in more detail previously (19), overnight-fasting blood samples were drawn to assess levels of blood lipids (i.e., high- and low-density cholesterol, triglycerides), insulin, and glucose. These were analyzed at the Department of Clinical Chemistry, Linköping University, Linköping, Sweden (ISO/IEC 17025) using appropriate methods (19). Fasting glucose and insulin values were used to calculate the homeostatic model for insulin resistance (HOMA-IR; i.e., fasting insulin (μU·L^−1^) × fasting glucose (mmol·L^−1^)/22.5) (27). Two repeated measures of systolic and diastolic blood pressure (in a sitting position after a 5-min rest) were performed using an electric sphygmomanometer (ProBP 3400 series; WelchAllyn, Skaneateles Falls, NY). In case of considerable difference between the measurements (>10 mm Hg), a third measurement was performed, and the averages of systolic and diastolic blood pressure were used in the analyses. A metabolic syndrome (MetS) score was calculated as described previously (28), but omitting waist circumference because the women were pregnant, and instead, FMI was included. Consequently, the MetS score was calculated as the standardized sum of the *z* scores of triglycerides, inverted HDL cholesterol, glucose, the average of systolic and diastolic blood pressure, and FMI.

### Statistical analysis

To investigate associations between different compositions of physical activity with body composition and cardiometabolic health, we conducted compositional data analysis. This type of analysis investigates the reallocation of time across behaviors over a specified continuum (i.e., 24 h) while lowering the risk of multicollinearity (12,29). One time-use composition was defined and included sleep, SB, LPA, and MVPA. First, isometric log ratios were calculated in sequential binary partition as previously proposed (12) and included as explanatory variables. The strength and direction of the association of each behavior relative to another (e.g., LPA relative to SB) with an outcome (e.g., body weight) is represented by gamma (*γ*) coefficients. The models’ coefficients were then used to predict the effect of reallocating time proportionally across behaviors (e.g., increasing MVPA while reducing the remaining behaviors) and pairwise (e.g., increasing MVPA while reducing SB) on the outcomes. Results are presented using pairwise time reallocation plots, showing the outcomes associated with reallocating time from one behavior to another (e.g., reallocating 30 min·d^−1^ to LPA from SB and sleep proportionally). The results can be interpreted as demonstrating the outcome associated with reallocating time between behaviors for a hypothetical average participant in our sample as all outcomes are relative to the mean behavior composition in the sample. For the cross-sectional analyses, we fitted an unadjusted and adjusted model (i.e., adjusted for age, parity (0 vs ≥1) and education level (university degree vs no university degree)). Two models (crude and adjusted) were fitted for the longitudinal analysis. In the crude model, the change in the outcome from gestational weeks 14 to 37 was included as the dependent variable, and the isometric log ratios for the movement behaviors at gestational week 14 (i.e., sleep, SB, LPA, and MVPA) together with the change in these isometric log ratios from gestational weeks 14 to 37 and the outcome at gestational week 14 as independent variables. The adjusted models were additionally adjusted for age, parity (0 vs ≥1), education level (university vs no university degree), and group allocation (intervention vs control). To assess the robustness of our findings, we also conducted sensitivity analyses. First, the Swedish Healthy Eating Index score in gestational weeks 14 and 37 was added to the adjusted model in the cross-sectional and longitudinal analyses, respectively, to examine whether diet influenced the derived estimates. Second, the models were also rerun excluding women with less than 4 valid days of accelerometer data (*n* = 8 in gestational week 14, *n* = 10 in gestational week 37) and only including women in the control group (*n* = 123), and results remained similar (data not shown). As provided elsewhere (18), the HealthyMoms trial was powered for the primary outcome (i.e., gestational weight gain), and in this analysis, our sample size (*n* = 242) would provide 80% power (two-tailed, *α* = 0.05) to detect a standardized regression coefficient of 0.18. Statistical analyses were performed using the statistical software R version 4.0.3 (R Foundation for Statistical Computing) and two-sided *P* values <0.05 were considered statistically significant.

## RESULTS

### Participant Characteristics

The participating women in this study (*n* = 272) were, on average (SD) 31 (4) yr of age, 77% (*n* = 209) had a university degree, 56% (*n* = 153) were nulliparous, and the average (SD) prepregnancy BMI was 23.5 (3.8) kg·m^−2^. Moreover, prepregnancy, 71% (*n* = 195) of the women had normal weight, 21% (*n* = 58) had overweight, 6% (*n* = 16) had obesity, and 2% (*n* = 4) had underweight, and 2% (*n* = 6) smoked. As reported previously, 242 women completed both measurements in this study (gestational weeks 14 and 37) and baseline characteristics (age, educational attainment, parity, prepregnancy BMI) as well as gestational weight gain and changes in movement behaviors for these women were similar to the whole sample (*n* = 273).

### Movement Behaviors, Body Composition, and Cardiometabolic Health Throughout Pregnancy

**FIGURE 1:**
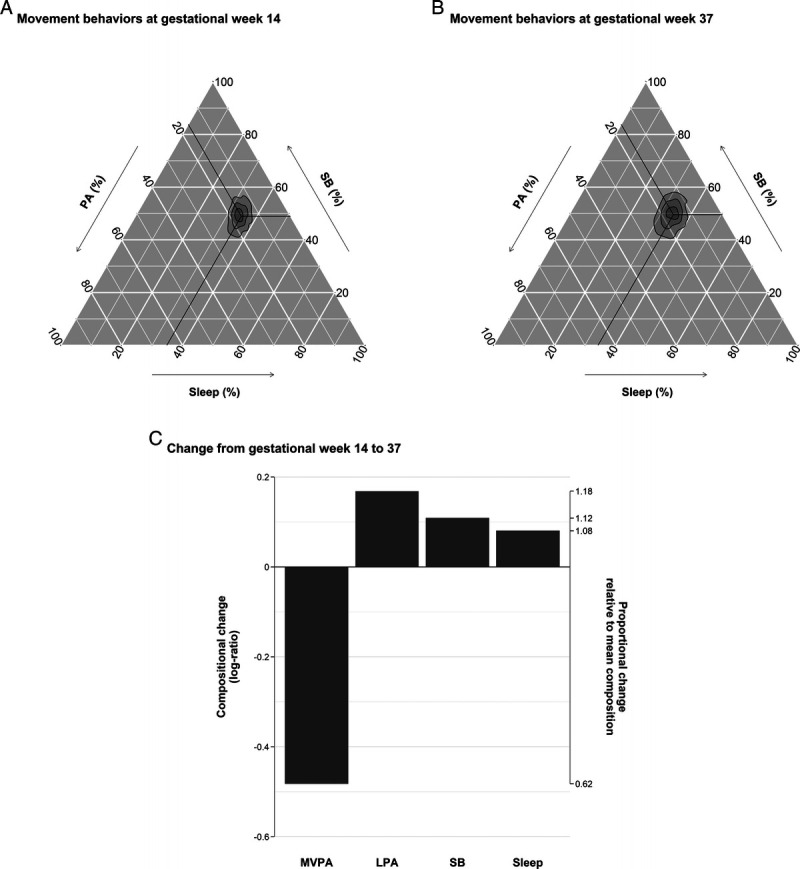
Distribution of movement behaviors at gestational weeks 14 and 37. A, Movement behaviors of HealthyMoms participants on a ternary plot, showing sleep, SB, and physical activity (PA) (PA combines LPA and MVPA). The crosshair marks the compositional mean at (A) gestational week 14 (i.e., MVPA: 32 min·d^−1^, LPA: 198 min·d^−1^, SB: 693 min·d^−1^, sleep: 493 min·d^−1^) and (B) gestational week 37 (i.e., MVPA: 18 min·d^−1^, LPA: 210 min·d^−1^, SB: 699 min·d^−1^, sleep: 484 min·d^−1^). Concentric rings represent the 25%, 50%, and 75% confidence regions for the data. B, Movement behaviors of HealthyMoms participants on a ternary plot, showing sleep, SB, and PA at gestational week 37. Concentric rings represent the 25%, 50%, and 75% confidence regions. C, Compositional change in MVPA, LPA, SB, and sleep with respect to the overall mean time composition. The left axis gives the log-ratio value, and the right axis displays the actual proportion relative to the mean composition (e.g., 1.25 means 1.25 times the compositional mean or a proportion higher by 25%).

Figure 1 shows the distribution of movement behaviors (i.e., sleep, SB, LPA, and MVPA) in early and late pregnancy (gestational weeks 14 and 37). The composition of movement behaviors was similar in early (Fig. 1A) and late pregnancy but with greater variation in late pregnancy (Fig. 1B). Regarding the change in the individual movement behaviors (Fig. 1C), the proportion of MVPA decreased by 38% from the sample average between early and late pregnancy, whereas time in the other movement behaviors increased (sleep, 8%; SB, 12%; and LPA, 18%, respectively). Table 1 presents data on body composition and cardiometabolic health at the two time points. As expected, body composition and cardiometabolic health indicators were generally higher in late pregnancy (*P* < 0.001).

**TABLE 1:** Body composition and cardiometabolic health in early (gestational week 14) and late (gestational week 37) pregnancy.

	Early Pregnancy (*n* = 273)	Late Pregnancy (*n* = 242)
Body composition		
Weight (kg)	67.3 (11.3)	77.6 (11.5)*
Height (m)	1.67 (0.06)	1.67 (0.06)
BMI (kg·m^−2^)*^a^*	24.1 (3.8)	27.8 (3.7)*
Fat mass (%)*^b^*	31.7 (7.3)	31.8 (6.2)
Fat mass (kg)*^b^*	22.0 (8.9)	25.2 (8.4)*
Fat-free mass (kg)*^b^*	45.4 (4.7)	52.5 (5.2)*
FMI (kg·m^−2^)*^b^*	7.9 (3.1)	9.0 (2.9)*
FFMI (kg·m^−2^)*^b^*	16.2 (1.3)	18.8 (1.5)*
Cardiometabolic health indicators		
Glucose (mmol·L^−1^)^*c*,*d*^	4.8 (0.3)	4.7 (0.4)*
Insulin (μIU·L^−1^)^*c*,*d*^	6.4 (2.9)	10.8 (5.0)*
HOMA-IR*^d^*	1.4 (0.7)	2.3 (1.2)*
Systolic blood pressure (mm Hg)	108 (9)	111 (10)*
Diastolic blood pressure (mm Hg)	70 (6)	73 (7)*
Total cholesterol (mmol·L^−1^)*^d^*	4.7 (0.6)	6.7 (1.0)*
Triglycerides (mmol·L^−1^)*^d^*	1.0 (0.4)	2.6 (0.9)*
HDL cholesterol (mmol·L^−1^)*^d^*	2.0 (0.3)	2.0 (0.4)*

Values are reported as mean (SD) for continuous variables or *n* (%) for categorical variables.

*Statistically significant from corresponding values in gestational week 14 (*P* < 0.001).

*^a^n* = 241 in gestational week 37.

*^b^n* = 240 in gestational week 37.

*^c^n* = 272 in gestational week 14.

*^d^n* = 236 in gestational week 37.

### Cross-Sectional Associations in Early Pregnancy

**FIGURE 2:**
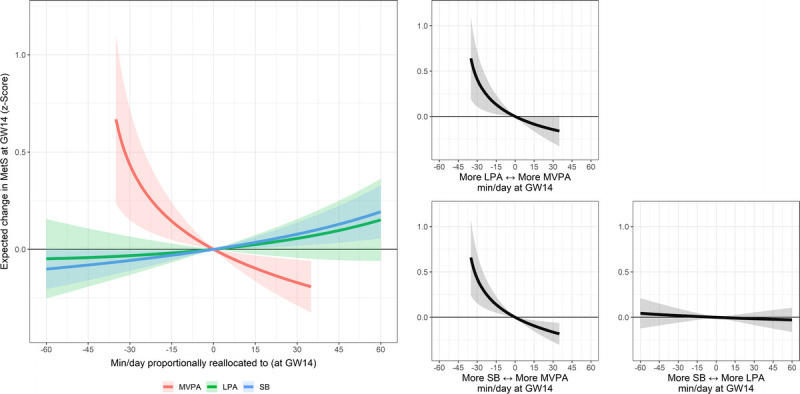
Cross-sectional associations of MVPA, LPA, SB, and sleep relative to the other behaviors in gestational week (GW) 14 with MetS score in GW14. The *colored lines* represent the effect of increasing one behavior while proportionally reducing the others (e.g., increasing MVPA while decreasing LPA, SB, and sleep). The *black line* represents the effect of increasing one behavior while proportionally reducing another (e.g., increasing MVPA while decreasing SB). Models are adjusted for maternal age, parity (0 vs ≥1), and education level (university vs no university degree).

The cross-sectional associations of movement behaviors with body composition and cardiometabolic health in early pregnancy (gestational week 14) are presented in Table S1 (see Supplemental Digital Content 2, Appendix, http://links.lww.com/MSS/C705). The results showed that reallocating time to LPA from SB and sleep in early pregnancy was associated with lower body weight (adjusted *γ* = −5.959, *P* = 0.047) and lower HOMA-IR (all *γ* ≤ −0.495, *P* ≤ 0.047) at the same time point. Moreover, reallocating time to MVPA from LPA, SB, and sleep was associated with lower MetS score (all *γ* ≤ −0.343, all *P* ≤ 0.002). The results remained after additional adjustment for diet quality (see Table S2, Supplemental Digital Content 2, Appendix, http://links.lww.com/MSS/C705). The dose–response curves relative to increasing one behavior while proportionally reducing the others (e.g., increasing MVPA while reducing LPA, SB, and sleep) and pairwise reallocation plots illustrating the effect size of replacing one behavior with another are shown in Figure 2 and Figures S1–S8 (see Supplemental Digital Content 2, Appendix, http://links.lww.com/MSS/C705). For example, reallocating 10 min·d^−1^ to MVPA from LPA, SB, and sleep was associated with lower MetS score (−0.07 SD; 95% confidence interval (CI), −0.12 to −0.03) in early pregnancy. Correspondingly, reallocating 10 min·d^−1^ from MVPA to SB showed an association in the opposite direction (0.09 SD; 95% CI, 0.04 to 0.14; Fig. 2) at this time point.

### Longitudinal Associations

#### Physical activity in early pregnancy and outcomes in late pregnancy

**FIGURE 3:**
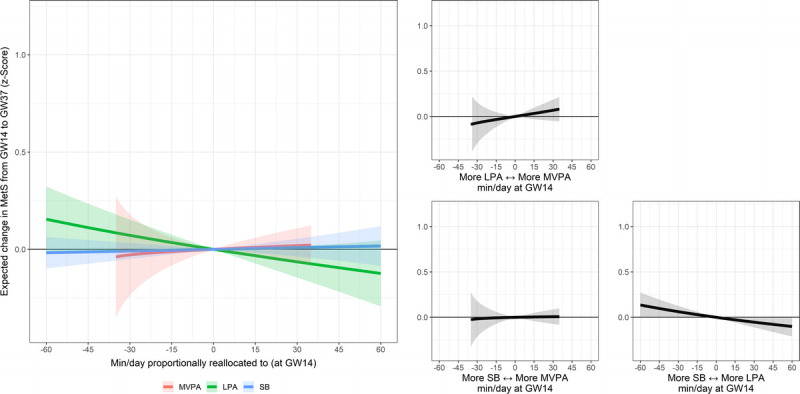
Longitudinal associations of MVPA, LPA, SB, and sleep relative to the other behaviors in gestational week (GW) 14 with MetS score in GW37. The *colored lines* represent the effect of increasing one behavior while proportionally reducing the others (e.g., increasing MVPA while decreasing LPA, SB, and sleep). The *black line* represents the effect of increasing one behavior while proportionally reducing another (e.g., increasing MVPA while decreasing SB). Model adjusted for movement behavior (i.e., MVPA, LPA, SB, and sleep) and outcome at baseline and follow and confounders (i.e., maternal age, parity (0 vs ≥1), education level (university vs no university degree), and group allocation (intervention vs control)).

Table S3 (see Supplemental Digital Content 2, Appendix, http://links.lww.com/MSS/C705) shows the associations of movement behaviors in early pregnancy (gestational week 14) with body composition and cardiometabolic health in late pregnancy (gestational week 37) assessed using compositional data analysis. Reallocating time to LPA from SB and sleep in early pregnancy was associated with lower FMI (adjusted *γ* = −0.668, *P* = 0.028), glucose levels (all *γ* ≤ −0.219, all *P* ≤ 0.043), HOMA-IR (all *γ* ≤ −0.619, all *P* ≤ 0.016), and MetS score (all *γ* ≤ −0.410, all *P* ≤ 0.040) in late pregnancy. These associations were adjusted for (i.e., were independent of) the change in behaviors throughout pregnancy. Results remained the same after additional adjustments for diet (see Table S4, Supplemental Digital Content 2. Appendix, http://links.lww.com/MSS/C705). Analyses with additional adjustment for diet also showed statistically significant associations between higher levels of LPA relative to SB and sleep in early pregnancy and body weight (γ = −2.086, *P* = 0.040) in late pregnancy. The dose–response curves relative to increasing one behavior while proportionally reducing the others (e.g., reallocating time to MVPA from LPA, SB, and sleep) and the pairwise reallocations plots illustrating the effect size of replacing one behavior with another in early pregnancy over the outcomes in late pregnancy are shown in Figure 3 and Figures S9–S16 (see Supplemental Digital Content 2, Appendix, http://links.lww.com/MSS/C705). For example, replacing 30 min·d^−1^ of SB with LPA in early pregnancy was associated with a decrease in MetS score (−0.05 SD; 95% CI, −0.11 to 0.00) in late pregnancy (Fig. 3).

#### Changes in movement behaviors throughout pregnancy and outcomes in late pregnancy

**FIGURE 4:**
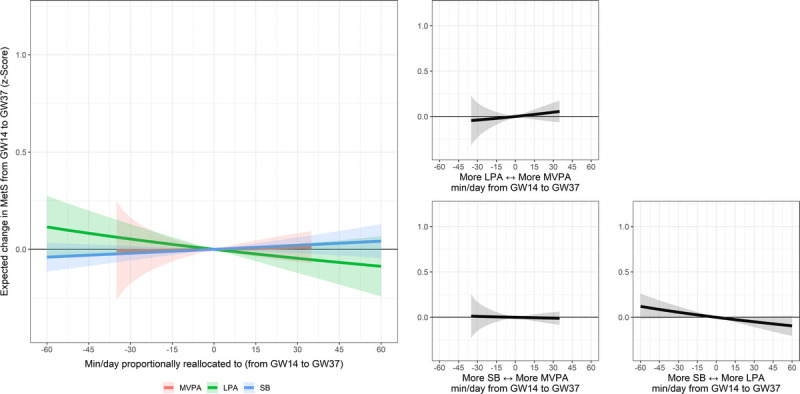
Longitudinal associations of change in MVPA, LPA, SB, and sleep relative to the other behaviors between gestational weeks (GW) 14 and 37 with MetS score in GW37. Each *colored line* represents the effect of increasing one behavior while proportionally reducing the others (e.g., increasing MVPA while decreasing LPA, SB, and sleep). Each *black line* represents the effect of increasing one behavior while proportionally reducing another (e.g., increasing MVPA while decreasing SB). Model adjusted for movement behavior (i.e., MVPA, LPA, SB, and sleep) and outcome at baseline and follow and confounders (i.e., maternal age, parity (0 vs ≥1), education level (university vs no university degree), and group allocation (intervention vs control)).

Table S5 (see Supplemental Digital Content 2, Appendix, http://links.lww.com/MSS/C705) reports the results for the associations between the change in behaviors from early to late pregnancy (gestational weeks 14–37) and outcomes in late pregnancy. The corresponding dose–response curves and pairwise reallocation plots are presented in Figure 4 and Figures S17–S24 (see Supplemental Digital Content 2, Appendix, http://links.lww.com/MSS/C705). Overall, as shown in Table S5 (see Supplemental Digital Content 2, Appendix, http://links.lww.com/MSS/C705), changes in movement behaviors from early to late pregnancy were not associated with body weight, body composition, glucose, HOMA-IR, or MetS score in late pregnancy. Results also remained essentially the same after additional adjustments for diet (see Table S4, Supplemental Digital Content 2, Appendix, http://links.lww.com/MSS/C705). Increasing time spent in MVPA while decreasing LPA, SB, and sleep throughout pregnancy was associated with a statistically significantly higher systolic (all *γ* ≤ 2.415, all *P* ≤ 0.010) and diastolic blood pressure (all *γ* ≤ 1.501, all *P* ≤ 0.041) in late pregnancy. However, as illustrated by the pairwise reallocation plots (see Figs. S23 and S24, Supplemental Digital Content 2, Appendix, http://links.lww.com/MSS/C705), increasing MVPA in favor of SB (+10 min·d^−1^) throughout pregnancy was associated with a very minor increase in systolic (0.40 mm Hg; 95% CI, 0.10 to 0.71 mm Hg) and diastolic blood pressure (0.24 mm Hg; 95% CI, 0.02 to 0.45 mm Hg) in late pregnancy. Finally, increasing LPA by 30 min·d^−1^ in favor of SB between early and late pregnancy was associated with a slightly lower FMI (−0.08; 95% CI, −0.16 to −0.00) in late pregnancy (see Fig. S19, Supplemental Digital Content 2, Appendix, http://links.lww.com/MSS/C705).

## DISCUSSION

### Main findings

This study is the first to investigate associations between movement behaviors with body composition and cardiometabolic health in early and late pregnancy accounting for the 24-h continuum. Overall, we observed that reallocating time to LPA from SB and sleep or to MVPA from LPA, SB, and sleep was associated with lower weight and more favorable cardiometabolic health in early pregnancy. In contrast, only LPA in early pregnancy seems to be beneficial for body composition and cardiometabolic health in late pregnancy. Finally, our results suggest that changes in movement behaviors between early and late pregnancy are of less importance for body composition and cardiometabolic health in late pregnancy.

### Comparison with previous studies

Our results showed that reallocating time to MVPA and LPA was associated with improved MetS score (including triglycerides, HDL, blood pressure, glucose levels, and FMI) and lower body weight and HOMA-IR, respectively, in early pregnancy. To the best of our knowledge, this is the first study to investigate associations between movement behaviors taking the 24-h continuum into account with body composition and cardiometabolic health in pregnancy. Moreover, few studies have investigated the associations between individual movement behaviors and body composition and metabolic health using objective methods, and these have mainly focused on outcomes in late pregnancy (e.g., [14–16,30–32]). Nevertheless, our results are consistent with a study by Loprinzi et al. (14) (*n* = 206), which found that women who engaged in higher levels of LPA and MVPA (objectively measured) had lower diastolic blood pressure and higher HDL cholesterol, respectively. In detail, our findings showed that reallocating as little as 10 min·d^−1^ to MVPA from LPA, SB, and sleep in early pregnancy was associated with a 0.07 SD decrease in MetS score. Moreover, our results are also consistent with previous studies that have investigated beneficial associations of objectively measured physical activity and body composition (e.g., BMI) and cardiometabolic health (e.g., glucose, insulin, HOMA-IR, and systolic and diastolic blood pressure) in adults using compositional data analysis (e.g., [12,33–36]). Although physiological changes (e.g., increased cardiac output) has been initiated and the body is preparing for the increased demands of the growing fetus, these results might reflect the nonpregnant state and lifestyle before pregnancy. Altogether, this indicates that MVPA, which is considered an essential part of a healthy lifestyle, as well as LPA are of importance for metabolic health also in early pregnancy.

In contrast to early pregnancy, late pregnancy is characterized by more pronounced physiological changes (e.g., decreased insulin sensitivity) (37), and thus, our results should be compared with previous longitudinal studies in pregnancy. Although we found that accumulating more LPA while reducing time spent in SB and sleep was associated with lower body weight in early pregnancy, we only observed a weak association with lower FMI in late pregnancy. Previous studies in pregnant women using objective methods to assess physical activity have shown conflicting results. To illustrate, Van Poppel et al. (15) (*n* = 24) found no associations between time spent in MVPA in early pregnancy or changes throughout pregnancy with weight gain, whereas Mizgier et al. (16) found that women engaging in more moderate physical activity (>21 min·d^−1^) in the second half of pregnancy (*n* = 28) had a reduced risk of excessive gestational weight gain compared with women who performed less (*n* = 29). Moreover, we found that accumulating more LPA in early pregnancy was associated with more favorable cardiometabolic health (i.e., lower HOMA-IR, glucose levels, and MetS score) in late pregnancy, independent of the change in physical activity throughout pregnancy. Similarly, previous studies in pregnant women have shown higher levels of MVPA in early pregnancy (<20 wk) to be associated with reduced insulin response (15) and lower insulin release (31). Furthermore, our results showed no statistically significant associations between change in movement behaviors between early and late pregnancy and the integrated MetS score considering several cardiometabolic risk factors or HOMA-IR in late pregnancy. This is in line with the results from Dieberger et al. (31) (*n* = 232), who found no associations with changes in MVPA throughout pregnancy and glucose and insulin levels. In contrast, Van Poppel et al. (15) (*n* = 24) found that women with a larger decrease in MVPA throughout pregnancy had higher fasting insulin levels and decreased insulin sensitivity in late pregnancy compared with women with smaller decreases or increases in MVPA (15). Potential explanations for the conflicting results could be, for example, differences in study characteristics and sample size as well as the time for assessment of physical activity and outcomes. However, both Van Poppel et al. (15) and Dieberger et al. (31) included women with overweight and obesity and an increased risk of gestational diabetes (compared with healthy women from all BMI categories in our study), and outcome assessment in all three studies was conducted in the third trimester (35–37 wk compared with 32-wk gestation) with similar decrease in MVPA during pregnancy in all three studies (−33%, −28%, and −38%). Compared with our study, physical activity was assessed using hip-worn accelerometers (15,31). However, correlations between hip- and wrist-worn accelerometers have been shown to be moderate to high in pregnant women (38). More research in this area is needed to make solid conclusions; however, current evidence indicates that levels of physical activity in early pregnancy might be more important compared with the change in physical activity during pregnancy. Nonetheless, this statement should be considered with caution because a continuous monitoring of physical activity levels throughout pregnancy is required to understand the dynamics of physical activity in relation to body composition and cardiometabolic health in pregnant women.

In summary, the promotion of sufficient levels of physical activity in early pregnancy seems relevant for maintaining healthy body composition and cardiometabolic health indicators during the course of pregnancy; and more research is needed to elucidate whether the changes in physical activity during pregnancy are associated with body composition and cardiometabolic health in pregnant women. Moreover, although moderate physical activity has been the focus of physical activity guidelines (8) and most previous studies have focused on moderate or MVPA (e.g., [15,16,31]), our results indicate that LPA is important for cardiometabolic health in late pregnancy. This can be considered positive because it might be easier to increase LPA compared with MVPA in pregnancy. Thus, in addition to what is emphasized in the current recommendations (i.e., increasing moderate physical activity) (8), women may be encouraged to also engage in LPA.

### Strengths and limitations

This study has several strengths including the relatively large sample of pregnant women that were measured using objective and standardized methods to assess physical activity (i.e., accelerometry), body composition (i.e., air-displacement plethysmography), and cardiometabolic health (e.g., fasting blood samples). Furthermore, exposure and outcome measures were assessed at two time points (i.e., in early and late pregnancy) enabling both cross-sectional and longitudinal analyses. Finally, the use of compositional data analysis accounts for the multicollinearity of accelerometer-derived physical activity data (12,29). The study also has limitations to acknowledge. The majority of women in our sample had a university degree (around 77%), which might somewhat decrease generalizability; however, our sample included women from all BMI categories with variation in both exposure and outcome variables. Moreover, physical activity tends to change during the course of pregnancy because some activities are not suitable in late pregnancy. Thus, women who regularly engaged in, for example, running as a means of engaging in MVPA in early pregnancy might choose other activities (e.g., bicycling or resistance training) in late pregnancy instead, which the accelerometer cannot capture (39). However, self-reported questionnaires to assess levels of physical activity have been shown to overestimate MVPA (40) and to have low precision (large absolute error and wide limits of agreement) for measuring SB in pregnancy (41).

### Clinical and public health relevance

One very interesting observation in this study is that reallocation of SB to LPA was associated with lower FMI and more favorable cardiometabolic health in late pregnancy. Previously, the focus has often been on increasing MVPA because pregnant women have been observed to have low adherence to the current recommendations of 150-min moderate physical activity per week (6,9) and often decline their levels of physical activity throughout pregnancy. However, it may be challenging to maintain MVPA on a high level as the pregnancy advances because of a higher body weight and potential pregnancy complications such as pelvic pain. In contrast, encouraging LPA in favor of SB also in late gestation might be easier to achieve. Moreover, our results indicate that efforts to promote LPA and MVPA in early pregnancy might be more important than maintaining the same level of physical activity in late pregnancy. The associations observed were only fairly strong, and it is likely to have public health relevance but may be of clinical relevance as well. However, more studies are needed to corroborate these findings, and future studies should also include more sedentary women and longer monitoring of movement behaviors throughout all stages of pregnancy. Altogether, our results support that it is important to set a foundation for a healthy pregnancy early on, or even before conception, and that LPA may also be a key target for health promotion in pregnant women.

## CONCLUSIONS

Our results demonstrated that reallocating time to LPA from SB and sleep, or to MVPA from LPA, SB, and sleep, in early pregnancy was associated with lower weight and more favorable cardiometabolic health at the same time point. In contrast, LPA in early pregnancy seems to be beneficial for body composition and cardiometabolic health indicators in late pregnancy. Furthermore, the change in behaviors throughout pregnancy was not associated with weight, body composition, and MetS score in late pregnancy. Our results indicate that interventions to promote healthy weight gain and cardiometabolic health in pregnancy may also encourage LPA, which might be easier for pregnant women to perform.
